# Health literacy meets the life-course perspective: towards a conceptual framework

**DOI:** 10.1080/16549716.2020.1775063

**Published:** 2020-06-26

**Authors:** Helle Terkildsen Maindal, Jens Aagaard-Hansen

**Affiliations:** a Department of Public Health, Section for Health Promotion, Aarhus University, Aarhus, Denmark; b Health Promotion, Steno Diabetes Center Copenhagen, Copenhagen, Denmark; c SA MRC Developmental Pathways for Health Research Unit, Faculty of Health Sciences, University of the Witwatersrand, Johannesburg, South Africa

**Keywords:** Health promotion, public health, research

## Abstract

This paper presents a novel conceptual framework combining the concepts of health literacy and life-course to guide public health planning and research. Health literacy is a key competence that enables individuals to navigate health-care systems and health promotion activities. The life-course perspective places emphasis on how disease risk accumulates along the life trajectory from fetal life onwards, and how it can even pass from one generation to the next. Our conceptual framework illustrates how different domains of health literacy are required, and how the unequal distribution of health literacy may be influenced by social determinants at different times in the life-course. Thus, it is essential to disaggregate health literacy into sub-themes and analyse them as they unfold in a long-term life-course perspective. The suggested framework would allow these patterns to be mapped, thereby enabling public health planners to strategically target health literacy promotion programmes to the right population segments at the right time.

## Background

This paper explores the interface between the concepts of health literacy and the life-course and presents a conceptual framework that combines the two perspectives to guide strategic public health planning and health promotion research. As there are no existing data, it should be emphasized that the examples provided to illustrate the idea are based on hypothetical data.

### Health literacy

The pathways from educational achievement to a variety of health outcomes have been well understood for decades [1,2]. A vast body of studies links literacy and health, and the concept of health literacy has emerged from this work [3]. Health literacy is defined as ‘the combination of personal competencies and situational resources needed for people to access, understand, appraise and use information and services to make decisions about health’ [4]. Poor health literacy is a worldwide challenge. In the Shanghai Declaration on Promoting Health in the 2030 Agenda for Sustainable Development, WHO recognizes health literacy as a critical determinant of health and a vital component of efforts to reduce inequalities in health [5]. In Europe, one in three people face health literacy challenges [6], including individuals with mental health problems, where up to half lack basic health literacy skills [7].

The concept of health literacy is both content and context specific, and it is developed and expressed in relation to others [8]. However, there is now a move from the assessment of generic health literacy to the assessment of specific domains such as food literacy [9] and physical literacy [10]. Evidence for effective interventions to improve health literacy is still sparse [11]. This is particularly worrying for younger people. As children grow into adolescents, their critical health literacy skills mature alongside their cognitive development. There is also potential for them to discuss and prioritize health messages from a variety of sources and to make informed decisions on health actions. Rowlands et al. refer to this currently underexplored area in health literacy as the ‘teachable moment’, in which major life events (both positive and negative) provide opportunities for health-related learning [1,2]. Furthermore, there is also good evidence for the benefits of intergenerational learning, such as family learning and skill development within communities [1,2].

### The life-course perspective

For the past four decades, seminal research has highlighted the importance of the life-course perspective [12,13]. Building on the Developmental Origins of Health and Disease (DOHaD) hypothesis and epigenetic research, the life-course concept describes how positive and negative influences accumulate throughout the life-course, affecting an individual’s risk of a wide range of diseases and of socioeconomic and educational achievements (i.e. successful school performance) [14]. For example, harmful events occurring early in the life-course can lead to reduced cognitive ability, educational outcomes and lifetime earnings [15] and an increased risk of non-communicable diseases [16]. The outline of the life-course concept described above, is conceived within a biological/biomedical frame of mind. It is important to emphasize that apart from the biological perspective applied in this paper, there are alternative perspectives on the life-course concept from psychological or sociological points of view [17]. Thus, whereas elderly persons are peripheral to intergenerational transfer of risk within a biological perspective, they may constitute a very important resource as seen from a psycho-social perspective.

**Figure 1.**
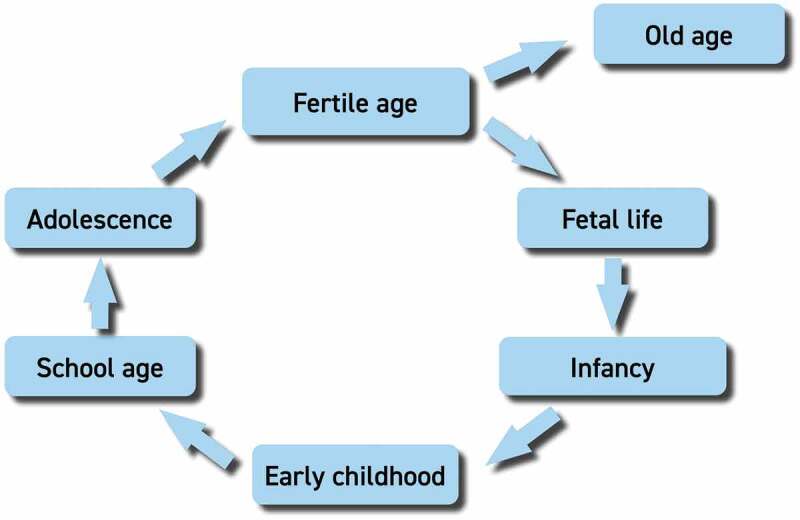
Circular representation of the main phases of the life-course, illustrating how each phase may influence the following one [19].

The life-course may be considered to span the period from birth to death. However, risk of disease accumulates not only throughout an individual’s life from the fetal stage onwards but is also passed on from one generation to the next [18]. Consequently, one can visualize the life-course as a circle incorporating each stage of life: fetal life, infancy, early childhood, school age, adolescence and fertile age (including the preconception period). Within this circle, positive and negative events at any stage of the life-course may have an impact on subsequent stages and even on following generations. This way of displaying the life-course emphasizes the ‘chicken and egg’ nature of the concept, which emphasizes the intergenerational causality chains where risk factors or interventions may have impact at later stages and generations. Old age is the exception, where the impacts of events are not transmitted to the next generation (Figure 1) [19]. Again it should be borne in mind that a sociological viewpoint implies a different view on old age citizens, who may actually constitute a powerful resource [17,20].

There is strong evidence that the experiences of early life, conceptualized as the first 1000 days of the life-course (including fetal life and first years of infancy), are associated with later health trajectories [21]. Some researchers argue that the current focus on the first 1000 days of the life-course should be expanded to a more holistic focus on the first 8000 days [22], while others advocate that more priority should be given to adolescent health [23]. Thus, it is critical to introduce effective interventions to promote good health from preconception to adolescence – the period during which an organism’s ability to adapt to its environment (i.e. its plasticity) is greatest and when the potential for affecting future generations is strongest [19]. The prominence of the life-course perspective is reflected in some of the key policy documents for the WHO European Region: the life-course is one of the four key priority areas in the Health 2020 policy document [24] and was the main focus of the Minsk Declaration, which stated that, ‘this approach adopts a temporal and societal perspective on the health of individuals and generations, including intergenerational determinants of health’ [20].

## Health literacy meets the life-course perspective

The concepts of both health literacy and the life-course have important and independent contributions to make to public health planning. Nevertheless, when the two perspectives are combined, new patterns and insights emerge that may inform public health planning and research. Health literacy is a key competence needed for individuals to benefit from health promotion activities and curative services. It can also be broken down into different domains (e.g. food or reproductive health), which can be incorporated into the design of specific health interventions. The life-course perspective adds a chronological dimension to our conceptual framework by spanning infancy, early childhood, school age, adolescence, reproductive age and old age. Focus on the accumulation of risk for various conditions over the life-course and across generations should be an essential underpinning of public health strategies and programmes [14].

**Figure 2.**
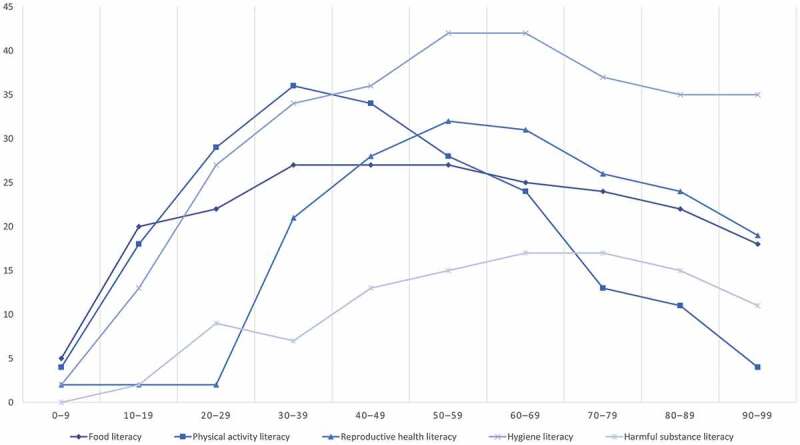
Hypothetical development of health literacy over the life-course stratified by health literacy domain. The x-axis indicates age intervals of decades. The y-axis indicates level of health literacy at an arbitrary scale of measurement (0–100%).

Health literacy is a skill that is acquired over most of the life-course based on family upbringing, learning from peers and formal schooling, though certain health conditions in old age may potentially reverse this trend. Low levels of health literacy are considered a risk factor for poor health outcomes, whereas improvement in health literacy along the life-course can be considered an asset for health promotion [25]. The resulting higher levels of parental health literacy can improve the health and subsequent health literacy of their infants, and can then be passed on to the next generation in a virtuous circle. The accumulation of health literacy along the life-course can be separated into temporal patterns for the different health literacy domains. Figure 2 outlines hypothetical trajectories for the development of health literacy along the life-course by different health literacy domains for a hypothetical population. Thus, within a given country or local community, one domain may predominate early in a child’s life (e.g. food literacy, where the curve has an early steep rise), while other domains may be less developed at the critical time of life (e.g. reproductive health literacy, which has been given a flatter trajectory).

If relevant data are available, this conceptual framework could be used to guide public health planning and ensure that resources are applied to the domains and age groups with the highest needs. For instance, health literacy related to reproductive health and harmful substances should be strongly promoted during adolescence. By combining health literacy with a life-course perspective, it would be possible to systematically determine whether all relevant domains have been addressed in a timely manner. In addition, this approach could encourage public health planners to consider new and hitherto neglected domains of literacy, such as essential elements of ‘parental (health) literacy’ [26], or ‘senior health literacy’, the latter of which may include fracture prophylaxis, dental health and the importance of sustaining social networks.

**Figure 3.**
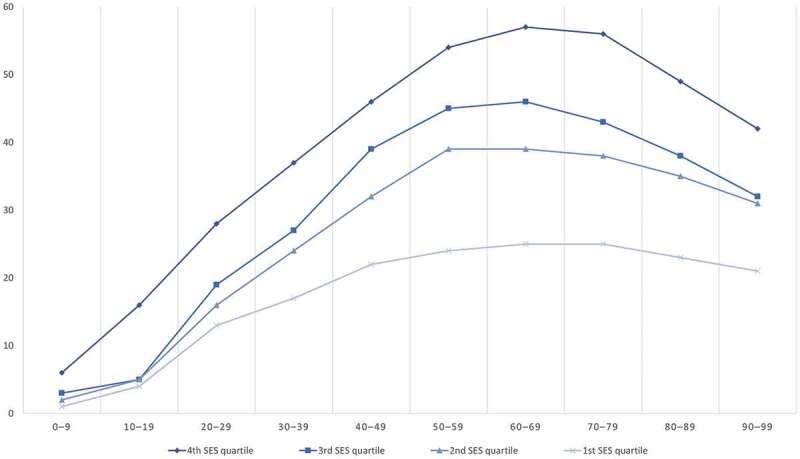
Hypothetical development of general health literacy over the life-course stratified by socioeconomic status (SES) quartiles. The x-axis indicates age intervals in decades. The y-axis indicates level of health literacy at an arbitrary scale of measurement (0–100%).

Trends in the different health literacy domains across the life-course are also influenced by various social determinants such as income, educational level, gender, ethnicity and religion. Figure 3 outlines how the hypothetical trajectories of general health literacy could be strongly influenced by socioeconomic status in a given population. By applying this framework to national data, public health planners may be able to identify specific gaps in health literacy among certain population segments (e.g. socioeconomic status strata), which would enable resources to be targeted to those most in need.

These hypothetical illustrations can inform future research priorities as well. In many cases, the equivalent real data are simply not available at national level, which limits the ability to target public health interventions to population groups where the needs are highest, thereby making them more cost-effective. Our conceptual framework has the potential to support more refined public health planning and the collection of relevant data is therefore a key priority.

Nevertheless, responsibility for population health literacy does not lie exclusively with the health services: the educational system must also act to ensure lifelong learning and local and national authorities should develop community health strategies. Thus, promoting high levels of health literacy throughout the life-course should be supported by cross-disciplinary teams of professionals across a variety of health promotion settings. Governments need to make coordinated efforts to increase health literacy throughout the life-course and to identify the optimal stages of learning for different age groups and population segments. Within the context of the Sustainable Development Goals (SDGs), health literacy incorporates SDG 3 on health as well as SDG 4 on education. In addition, although health literacy is an important factor, more structured public health measures are also needed to ensure healthy lives for all [27].

## Conclusions

Since the 1970ʹies, two concepts relevant to public health have rapidly developed. The notion of health literacy has become a key element in health promotion and has been refined to include separate domains such as food and physical activity literacy. The life-course perspective shows how risk accumulates both within an individual’s life-course and across generations. We have combined the two concepts into a conceptual framework that has the potential to inform public health planning and health promotion research. The conceptual framework offers public health planners a tool to apply a systematic approach to strengthening health literacy at population level. Use of this tool would ensure that appropriate domains of health literacy are efficiently made available at relevant phases of the life-course and that specific population segments are approached at the most ‘teachable’ moments. In addition, the framework may guide research agendas to provide the necessary data.

## Paper context

Both health literacy and the life-course perspectives have gained much attention in recent decades. The article suggests a conceptual framework that explores how a combination of the two may guide public health planning and research by disaggregating health literacy according to either domains or social determinants over time.
